# The effect of postgraduate training in smoking cessation care on the clinical practice of pulmonologists

**DOI:** 10.18332/tid/159795

**Published:** 2023-03-08

**Authors:** Pinar Bostan, Banu Salepci, Mehmet A. Uysal

**Affiliations:** 1 Faculty of Health Sciences, Istanbul Bilgi University, Istanbul, Turkey; 2 Department of Chest Diseases, Yeditepe University Kozyatagi Hospital, Istanbul, Turkey; 3 Yedikule Chest Diseases and Thoracic Surgery Hospital, University of Health Sciences Turkey, Istanbul, Turkey

**Keywords:** smoking cessation, postgraduate training, pulmonologist

## Abstract

**INTRODUCTION:**

Many studies have shown that training in smoking cessation care (SCC) is important for increasing the number and quality of delivered interventions by health professionals, and various training methods are available. The study aimed to identify the relationship between receiving training on SCC and the frequency of providing outpatient-based SCC among pulmonologists who were members of the Turkish Thoracic Society (TTS).

**METHODS:**

For this cross-sectional study, a self-administered online questionnaire-based survey was conducted on a group of active pulmonologists who were members of the TTS, between April and October 2019. The survey included questions about demographics, smoking status, participation in SCC training, and providing outpatient-based SCC.

**RESULTS:**

A total of 199 (53%) pulmonologists were actively taking part in outpatient-based SCC. Compared to those that were not providing outpatient-based SCC, median age, median time since graduation, and the number of non-academics, non-current smokers and recipients of smoking cessation care training were significantly higher in the group providing outpatient-based SCC (p<0.001, p<0.001, p=0.002, p=0.001, respectively). It was observed that having SCC training increased more than 6-fold the likelihood of providing outpatient-based SCC (AOR=6.45; 95% CI: 3.96–10.49; p<0.001).

**CONCLUSIONS:**

The most crucial obstacle in providing smoking cessation is healthcare workers not providing smoking cessation to smokers. It is worthwhile to devote more tasks and resources to training physicians on smoking cessation care since this may increase their effective involvement in tobacco cessation.

## INTRODUCTION

Tobacco use continues to be a significant contributor to the development of numerous serious diseases and premature mortality. Tobacco consumption causes more than 8 million deaths a year worldwide^1^. The WHO Framework Convention on Tobacco Control (WHO FCTC), which opened for signatures between 16 and 22 June 2003 in Geneva, is a set of guidelines that provide a foundation for implementing and managing tobacco control^2^. The MPOWER measures were developed by the WHO to help facilitate the framework and the measure ‘Offer help to quit smoking’ is one of the six strategies aimed at assisting in the execution of effective tobacco demand reduction programs at the country level^2^. There is a specific need to decrease the demand for tobacco through the promotion of education of health professionals and public awareness of the adverse effects of tobacco use, in addition to the implementation of nationwide smoking cessation campaigns^3-5^. The provision of support for quitting smoking is an essential component of any tobacco control policy. It is well established that many tobacco users prefer to quit, and tobacco users considerably increase their odds of successfully quitting with the support of cost-effective population-based interventions^6^.

Since millions of people consult with them, health practitioners are uniquely positioned to tackle the epidemic of tobacco use. The role and image of health professionals are essential factors in promoting a tobacco-free lifestyle^7^. Studies conducted as far back as the 1970s have indicated that physician interventions are beneficial in helping people to quit smoking^8-11^. A meta-analysis by Stead et al.^12^ that comprised 41 trials and over 31000 participants indicated that even a brief intervention from a physician encourages individuals to quit smoking^12^. The interventions may vary from a brief intervention by simply asking and advising patients about smoking (3As brief intervention: Ask, Advice, Assess) to counselling (5As smoking cessation counselling: Ask, Advice, Assess, Assist, Arrange) by setting a quit date, scheduling follow-up appointments, providing of self-help materials for cravings, etc.^13^. Tobacco cessation training for health professionals has been shown to improve the professional performance of counselling in a systematic review published over a decade ago. A Cochrane Review of ten randomized controlled studies, on health professional smoking cessation training, found that those who got training were more likely to provide counselling, set a quit date, and organize follow-up^14^. So numerous studies have demonstrated that providing training on smoking cessation care to health professionals is an important strategy for enhancing the number and quality of quitting interventions offered by health professionals, for which a variety of training options exist. Smoking cessation education programs are available at both undergraduate (for medical students) and postgraduate levels of education. At the postgraduate level, the programs are typically delivered in the form of vocational training or continuing medical education (CME)^15^.

In this study, we focused on postgraduate education, either as part of residency training or as part of CME directly applied to practicing pulmonologists (chest physicians). We aimed to identify the relationship between receiving training on SCC and the frequency of providing outpatient-based smoking cessation care among pulmonologists who were members of the TTS.

## METHODS

### Study design and participants

For this cross-sectional study, a self-administered online questionnaire-based survey was conducted, between April and October 2019, among a group of pulmonologists who were members of the Turkish Thoracic Society. The TTS is an organization for healthcare professionals, akin to various pulmonology associations worldwide, and most of its members are pulmonologists.

The findings of this study were produced by reanalyzing part of the data obtained from an overarching TTS scientific research project entitled ‘Knowledge, attitudes, and behavior of the pulmonologist members of TTS towards tobacco and new tobacco products’. The study protocol was approved by the TTS Scientific Project Committee. After a pre-test, the TTS secretariat emailed a link to a self-administered online questionnaire on Survey Monkey (surveymonkey.com) containing a written informed consent form to 2941 pulmonologist members of TTS. Weekly reminders were made to boost the number of participants. As we could not confirm if the e-mails were received and read by all these members, we estimated the response rate as the number of total completed surveys divided by the number of registered TTS pulmonologists. The questionnaire asked about gender, age, medical school graduation date, academic position, smoking status, the existence and the source of training on smoking cessation care (SCC), and about the presence of providing outpatient-based SCC. Outpatient-based SCC includes providing both counselling and smoking cessation medication in a dedicated smoking cessation outpatient clinic that is offered as an outpatient health service in Turkey. The categorical variables were dichotomized, e.g. academic position was dichotomized as having or not having any academic employment.

The WHO classification system was used to determine smoking status^16^. Individuals who had smoked for at least six months in their lives and who were smoking continually at the time of the survey were categorized as ‘current smokers’. Ex-smokers (smoked for at least 6 months during their lifetime but not within the 6 months prior to the survey), recent quitters (smoked for at least 6 months during their lifetime but not within the 6 months prior to the survey), and never smokers (never smoked or had smoked for less than 6 months or fewer than 100 cigarettes during the survey) were classified as ‘non-current smokers’.

### Statistical analysis

Descriptive analyses were performed using frequency, percentage, mean with standard deviation, median with interquartile range (IQR) or simple range (min–max). Comparisons were performed concerning various classifications of responders. The Pearson’s chi-squared test assessed differences in the distribution of categorical variables. For continuous data analysis, the independent samples t-test was used to compare variables with normal distribution. The non-normally distributed variables were tested with the Mann–Whitney U test. After adjusting for relevant confounders identified with univariate analysis, the association between providing outpatient-based SCC and having received SCC training was evaluated using logistic regression. Calculated odds ratios (AOR) and 95% confidence intervals (95% CI) were used to evaluate the strength of the associations. A p<0.05 was regarded as statistically significant. All analyses were performed using SPSS for Windows (version 26.0; SPSS Inc., Chicago, IL, USA).

## RESULTS

Between April and October 2019, all pulmonologists who were members of the TTS were sent the online questionnaire, and 374 responded. Pulmonologists who answered the questionnaire were 12.7% (374/2941) of the registered pulmonologist members of TTS. When responders were analyzed, 61.8% (n=231) were females, and the median age was 43 years (range: 34–52). Our study group did not differ from the overall group of TTS -registered pulmonologists in terms of age and gender (p=0.637; p=0.883, respectively). However, the proportion of physicians who were academics was significantly higher among the respondents (p<0.001) (Table 1).

**Table 1:** Comparison of demographic properties of all pulmonologist members of the Turkish Thoracic Society and the responders of the self-administered online questionnaire-based survey, April–October 2019

	*Pulmonologists (N=2941) n (%)*	*Responders (N=374) n (%)*	*p*
**Gender**			0.883
Female	1828 (62.2)	231 (61.8)	
Male	1113 (37.8)	143 (38.2)	
**Age** (years)*, median (IQR)	43 (35–51)	43 (34–52)	0.637
**Academic position**			<0.001
Yes	1247 (42.4)	197 (52.7)	
No	1694 (57.6)	177 (47.3)	

IQR: interquartile range.

* Mann-Whitney U test.

Among the respondents, 195 (52.1%) had received SCC training, and 133 (68.2%) of those who were trained had been trained in the courses organized by TTS, including central/regional courses, congress courses, certification training carried out with the Ministry of Health, and as part of the ‘Learning ground for quitting smoking’ project, all of which had been conducted within the scope of CME. Apart from these, 17 individuals (8.7%) had received SCC training (vocational training) during their residency, 24 (12.3%) reported that they had attended the Ministry of Health Certification training directly, and the remaining 21 (11%) were those who had benefited from the education of different associations (n=14) and those with no data (n=7).

The comparison of those with and without SCC training showed that, in individuals with SCC training, the median age was higher (46 vs 38 years, respectively, p<0.001), and the time that had passed since graduation was longer (23 vs 14 years, respectively, p<0.001). In addition, it was found that the percentage of non-academics (54.8% vs 39.1%, p=0.002) and those who were non-current smokers (95.4% vs 84.9%, p=0.001) were significantly higher in those with SCC training (Table 2).

**Table 2:** Comparison of characteristics of the pulmonologists with and without training in smoking cessation care (SCC)^a^, April–October 2019

*Characteristics*	*With SCC training n (%)*	*Without SCC training n (%)*	*Overall n (%)*	*p*
**Total**	195 (52.1)	179 (47.8)	374 (100)	
**Gender**				0.6
Female	118 (60.5)	113 (63.1)	231 (61.8)	
Male	77 (39.5)	66 (36.9)	143 (38.2)	
**Age** (years)*, median (IQR)	46 (39–55)	38 (31–50)	43 (34–52)	**<0.001**
**Time since graduation** (years)*, median (IQR)	23 (15–31)	14 (7–25)	19 (2–52)	**<0.001**
**Academic position**				**<0.001**
No	107 (54.8)	70 (39.1)	177 (47.3)	**0.002**
Yes	88 (45.2)	109 (60.9)	197 (52.7)	
**Smoking status**				
Current smokers	9 (4.6)	27 (15.1)	36 (9.6)	**0.001**
Non-current smokers	186 (95.4)	152 (84.9)	338 (90.4)	

a Among the responders of the self-administered online questionnaire-based survey.

* Mann-Whitney U test. IQR: interquartile range.

A total of 199 (53%) physicians were actively taking part in outpatient-based SCC. Compared to those not providing outpatient-based SCC, median age (45 vs 40 years, respectively, p<0.001), time since graduation (21 vs 15 years, respectively, p=0.001), number of non-academics (54.3% vs 39.4%, respectively, p=0.004), non-current smokers (93.5% vs 86.9%, respectively, p=0.003) and recipients of SCC training (73.4% vs 28.0%, respectively, p<0.001) were significantly higher among those providing outpatient-based SCC (Table 3).

**Table 3:** Comparison of the characteristics of pulmonologists who were and were not providing outpatient-based SCC^a^, April–October 2019

*Characteristics*	*Providing outpatient-based SCC n (%)*	*Not providing outpatient-based SCC n (%)*	*Overall n (%)*	*p*
**Total**	199 (53.0)	175 (47.0)	374 (100)	
**Gender**				
Female	124 (62.3)	107 (61.1)	231 (61.8)	0.8
Male	75 (37.7)	68 (38.9)	143 (38.2)	
**Age** (years)*, median (range)	45 (27–75)	40 (21–75)	43 (21–75)	**<0.001**
**Time since graduation** (years)*, median (range)	21 (2–52)	15 (2–48)	19 (2–52)	**0.001**
**Academic position**				**0.004**
No	108 (54.3)	69 (39.4)	197 (52.7)	
Yes	91 (45.7)	106 (60.6)	177 (47.3)	
**Smoking cessation care training**				
Received	146 (73.4)	49 (28.0)	195 (52.1)	**<0.001**
Not received	53 (26.6)	126 (72.0)	179 (47.9)	
**Smoking status**				**0.03**
Current smokers	13 (6.5)	23 (13.1)	36 (9.6)	
Non-current smokers	186 (93.5)	152 (86.9)	338 (90.4)	

a Among the responders of the self-administered online questionnaire-based survey.

* Mann-Whitney U test. SCC: smoking cessation care.

Parameters demonstrating a significant relation with providing outpatient-based SCC in univariate analysis, such as having SCC training, age, time since graduation, not having an academic position, and non-current smoking status were included in the logistic regression model. Logistic regression analysis with providing outpatient-based SCC as the dependent variable revealed that the other factors, except having SCC training, were not associated with the status of providing outpatient-based SCC (p=0.541, p=0.566, p=0.088, and p=0.469, respectively). It was observed that having SCC training increased by more than 6-fold the likelihood of providing outpatient-based SCC (AOR=6.4; 95% CI: 3.9–10.4, p<0.001) (Table 4).

**Table 4:** Logistic regression analysis for the association between providing outpatient-based SCC and having received training on SCC^a^, April–October 2019

*Variable*	*AOR*	*95% CI*	*p*
Age (years)	0.95	(0.84–1.06)	0.541
Time since graduation (years)	1.03	(0.91–1.18)	0.566
Being current smoker	0.74	(0.33–1.64)	0.469
Receiving SCC training	6.45	(3.96–10.49)	<0.001
Not having any academic position	1.49	(0.94–2.37)	0.088

a Among the responders of the self-administered online questionnaire-based survey.

SCC: smoking cessation care. AOR: adjusted odds ratio; adjusted for age, time since graduation, current/non-current smoker, receiving/not receiving SCC training, having/not having any academic position.

When the pulmonologists who were providing outpatient-based SCC were asked about their self-competence in providing SCC, the recipients of SCC training had a higher sense of competence in providing SCC than those without training (65.8% vs 41%, respectively, p=0.002). Lower percentage but a similar difference was observed among the pulmonologists who were not providing outpatient-based SCC in the comparison according to having received SCC training (38.8% vs 11.9%, respectively, p<0.001) (Table 5).

**Table 5:** The relationship between having received training on and feeling competent about providing SCC^a^, April–October 2019

		*With training n (%)*	*Without training n (%)*	*Overall n (%)*	*p*
**Pulmonologists providing SCC in the dedicated SCC outpatient clinic services**	‘Do you feel competent in providing SCC in your dedicated SCC outpatient clinic?’				
	Definitely yes	96 (65.7)	22 (41.5)	118 (59.3)	**0.002**
	Partially	49 (33.6)	28 (52.8)	77 (38.7)	
	Definitely no	-	3 (5.7)	3 (1.5)	
	No answer	1 (0.7)	-	1 (0.5)	
	Total	146 (100)	53 (100)	199 (100)	
**Pulmonologists providing SCC while routinely practicing in the chest diseases outpatient clinic services**	‘Do you feel competent in providing SCC to your patients who request smoking cessation support, while routinely practicing in your chest diseases outpatient clinic?’				
	Definitely yes	19 (38.8)*	15 (11.9)*	34 (19.4)	**<0.001**
	Partially yes	29 (59.2)	77 (61.1)	106 (60.6)	
	Definitely no	1 (3.0)	32 (25.4)	33 (20.0)	
	No answer	-	2 (1.6)	2 (1.1)	
	Total	49 (100)	126 (100)	175 (100)	

a Among the responders of the self-administered online questionnaire-based survey.

SCC: smoking cessation care.

* Mann-Whitney U test.

In the questionnaire, we addressed the following question to the 175 physicians who did not take part in outpatient-based SCC: ‘Can you spare enough time for your patients who request smoking cessation assistance while routinely practicing in your chest diseases outpatient clinic?’. The results revealed that 109 (62.3%) of the physicians answered ‘No’ to the question. When evaluated in subgroups, 27 (55.1%) of those with SCC training replied with ‘No’, and 82 (65.1%) in those without SCC training. There was no statistically significant difference between the two groups (p=0.2).

## DISCUSSION

In this self-administered online questionnaire-based survey, we investigated smoking cessation care (SCC) training and factors associated with providing outpatient based SCC among pulmonologists who were members of the TTS. It was determined that 53% of the pulmonologists who answered the questionnaire provided out-patient-based SCC, and 52.1% had received SCC training. According to logistic regression analysis, the only independent factor affecting outpatient-based SCC was SCC training. Our research is significant in terms of showing that the number of pulmonologists who provide outpatient-based SCC can increase in proportion to the education they receive on this topic. So, there is a potential for an increase in the number of physicians who provide smoking cessation care if more emphasis is placed on education regarding this topic.

In 2014, Pazarli et al.^17^ found a similar percentage (52.4%) of pulmonologist members of the TTS that had received SCC training among. The fact that this percentage is similar to ours indicates that the efforts in training them on SCC may be insufficient. This is true also in the group of physicians who attend to smoking-related diseases most frequently, even though Turkey is reported to be among those providing comprehensive tobacco cessation programs and implementing all MPOWER measures at the most comprehensive level^6^. It may indicate the necessity of devoting more tasks and resources to SCC training in addition to being more reachable by physicians.

Turkey signed the Framework Convention on Tobacco Control (FCTC) in 2005 and subsequently published the National Tobacco Control Program and Action Plans for different periods. In the ‘Smoking Cessation’ theme of these programs, it is strongly advised that all healthcare personnel and psychological counsellors should receive training on nicotine addiction, tobacco control, and smoking cessation techniques before graduation. In addition, this action plan also suggested that arrangements should be made to facilitate short interventions for clinicians throughout all clinical encounters, including primary care^18^. According to this plan, physicians should question the smoking status of all patients encountered, and if the patient is a smoker, the physician should provide options to help the patient to quit smoking. Pulmonologists have to be more sensitive about smoking cessation care, as a great proportion of the diseases dealt by these physicians are closely related to smoking. For this reason, the ‘Smoking Cessation Diagnosis and Treatment Consensus Report’ was published by the Tobacco Control Working Group of the TTS, in 2014^19^. In addition, ‘Smoking Cessation Care Training Courses’ are periodically organized by Chest Diseases Specialist Associations, especially TTS, and the Ministry of Health and the certificates given at the end of the courses are approved by the Ministry. Family physicians, public health physicians, practitioners, psychiatrists and especially pulmonologists attended these courses. The fact that only 51.2% of the respondents stated that they received training on SCC reveals the need to expand these training measures carried out by the Ministry of Health and specialty associations.

Since 2012, only those with this official certification mentioned above have been permitted to provide outpatient-based smoking cessation care. A small population (n=53) who began providing outpatient-based SCC before 2012 and lacked these training certificates were also included in the present study. Comparing this group of pulmonologists to those who received SCC training and certification, the proportion of pulmonologists who felt ‘certainly competent in providing SCC’ was significantly lower. However, the percentage (65.8%) in the group that received SCC training was different than expected, is also noteworthy. This result has been interpreted as a clear indication of the need for new regulations in SCC training and conditions for providing outpatient-based SCC to ensure that all trained physicians feel competent in this regard. Most pulmonologists cannot take the time to provide SCC to their patients, as demonstrated by the fact that 62.3% (n=109) of the pulmonologists answered ‘No’ to ‘Can you spare enough time for your patients who request smoking cessation care while routinely practicing in your chest diseases outpatient clinic?’, a question addressed to the 175 physicians who did not provide SCC in a dedicated SCC outpatient clinic. Moreover, it is also important to note that the difference between the groups with and without SCC training was not found to be statistically significant with respect to their response to this question. In other words, the fact that physicians received training was not found to be related to their ability to spare time for SCC while carrying out their duties in their routine chest disease outpatient clinics – which are extremely active clinics admitting a great number of individuals in Turkey. This result once again emphasizes the necessity of dedicated SCC outpatient clinic services. There is no question that every medical professional should make it a goal to give at least a brief intervention for patients who smoke. However, it is abundantly clear that it is nearly impossible to provide full SCC in the setting of any general or subspecialist outpatient clinic. There is a dose-response relationship between the intensity of counselling and its effectiveness, as shown by the fact that minimal duration counselling (up to 3 minutes) results in an abstinence rate of 13.4% at 6 months, low-intensity counseling (3–10 minutes) results in an abstinence rate of 16%, and higher intensity counseling (>10 minutes) results in an abstinence rate of 22%. This is significant when taking into consideration the fact that the amount of time spent providing SCC for smokers^11^. In a study comparing smoking cessation treatment practices across US healthcare systems, researchers discovered that Veterans Health Administration (VHA) healthcare providers had four times the odds of self-reported evidence-based smoking cessation treatment compared to academic health center (AHC) healthcare providers. The reason for the significant difference between the smoking cessation treatments provided by these healthcare systems was that the VHA had an outpatient-based smoking cessation treatment program^20^.

The providers of tobacco-related education to physicians may vary by country. Universities (39%) and a variety of other institutions, including residency training programs, hospitals, medical societies, health agencies, and non-governmental organizations, are the most prevalent providers of such programs worldwide^15^. Lack of training and expertise in smoking cessation has been recognized as a factor that discourages physicians from participating in SCC for their patients^21^, and such discouragement has been reported as a barrier to the conduct of SCC in other research^22,23^. Lack of training and competence can result in the use of ineffective tactics and the underuse of beneficial approaches in smoking cessation^24-27^. On the other hand, published research shows limited evidence that physicians who undergo SCC training achieve greater quit rates than those who do not^21,28^. In addition, there is a dearth of evidence proving which educational techniques are the most beneficial for enhancing physicians’ smoking-cessation skills. Interactive CME events that allow participants to practice skills through case discussions, role play, or hands-on sessions are more likely to result in a change in professional practice, according to an assessment of formal CME education^21^. In many countries, smoking cessation education programs are often offered ad hoc, with no regional or national strategy to ensure their availability and consistency^15^. New techniques and potential strategies are required to boost the availability and uptake of postgraduate education in smoking cessation by healthcare professionals and incorporate smoking cessation education into vocational courses for different disciplines.

Our results showed that pulmonologists who provided outpatient-based smoking cessation care were older, more experienced and mainly not smoking currently, non-academic physicians who received SCC training.

### Limitations

The main limitation of our study is that it was conducted among only the pulmonologists who are members of the same association. Secondly, the fact that the response rate was only 12.7% is a significant problem that could have altered the assessment results of the population (sampling and ascertainment bias). In relation, response and non-response bias may have also occurred, particularly with respect to factors such as time since graduation, SCC training status (individuals who were more aware of SCC or who had received training could have been more inclined to respond), the experience of SCC training (responses could have been skewed based on the perceived benefit from prior training), and academic status (academic and non-academic individuals may have responded unequally). Also, we did not determine the time since the last SCC training of the pulmonologists included in this study, and this characteristic may have influenced the results. Lastly, we were unaware of the personal reflections and motivations for providing outpatient-based SCC. Physicians may not provide outpatient-based SCC due to their constraints, such as perceived low efficacy of intervention effect on quitting smoking^29^, or because of their thoughts that they were not monetarily reimbursed for the time and effort they dedicated to smoking cessation care within the national health system.

## CONCLUSIONS

The most crucial obstacle to offering smoking cessation support, which is one of the pillars of tobacco control programs, is not achieving the projected rate of offering smoking cessation to smokers by healthcare workers. It is worthwhile to devote more tasks and resources to training physicians on smoking cessation care since it may potentially increase their involvement in tobacco control.

## Data Availability

The data supporting this research are available from the authors on reasonable request.
